# Noise edge pitch and models of pitch perception

**DOI:** 10.1121/1.5093546

**Published:** 2019-04-15

**Authors:** William M. Hartmann, Peter A. Cariani, H. Steven Colburn

**Affiliations:** Department of Physics and Astronomy, Michigan State University, 567 Wilson Road, East Lansing, Michigan 48824, USA; Hearing Research Center, Department of Biomedical Engineering, Boston University, 44 Cummington Street, Boston, Massachusetts 02115, USA

## Abstract

Monaural noise edge pitch (NEP) is evoked by a broadband noise with a sharp falling edge in the power spectrum. The pitch is heard near the spectral edge frequency but shifted slightly into the frequency region of the noise. Thus, the pitch of a lowpass (LP) noise is matched by a pure tone typically 2%–5% below the edge, whereas the pitch of highpass (HP) noise is matched a comparable amount above the edge. Musically trained listeners can recognize musical intervals between NEPs. The pitches can be understood from a temporal pattern-matching model of pitch perception based on the peaks of a simplified autocorrelation function. The pitch shifts arise from limits on the autocorrelation window duration. An alternative place-theory approach explains the pitch shifts as the result of lateral inhibition. Psychophysical experiments using edge frequencies of 100 Hz and below find that LP-noise pitches exist but HP-noise pitches do not. The result is consistent with a temporal analysis in tonotopic regions outside the noise band. LP and HP experiments with high-frequency edges find that pitch tends to disappear as the edge frequency approaches 5000 Hz, as expected from a timing theory, though exceptional listeners can go an octave higher.

## I. INTRODUCTION

### A. Background

In 1963, Békésy observed that an octave band of noise (400–800 Hz) produces two pitch sensations, one near each frequency edge of the noise spectral band. Békésy attributed the pitch sensations to lateral inhibition at the edges and compared them to Mach bands in vision. Small and Daniloff (1967) simplified Békésy's experiment by using lowpass (LP) noise bands and highpass (HP) noise bands. Their listeners adjusted the edge frequency of a noise band to produce a pitch an octave higher or lower than the pitch of a noise band with a standard edge frequency. Fastl (1971) performed pitch matching experiments in which the pitches elicited by LP and HP noise bands with sharp edges were matched by adjusting the frequency of a sine tone. In all of these early studies, the average matching frequencies were reported to be the same as the edge frequencies (e.g., Zwicker and Fastl, 1999), but later studies, as described below, showed pitch shifts where the matching frequencies deviate systematically from the edge frequencies.

In their work on binaural edge pitch, Klein and Hartmann (1981) recorded pitch matches for diotic LP and HP noise bands with sharp edges as a diotic analog to binaural edge pitch. Their pitch matching data consistently revealed pitch shifts away from the edge and into the noise. Thus, the pitch of a LP noise was found to be slightly below the edge frequency, and the pitch of a HP noise was found to be slightly above the edge frequency.

Klein and Hartmann generated their stimuli digitally in the frequency domain, leading to spectral edges with a 30-dB discontinuity at the edge frequency. These edges were much sharper than were available with the analog filters used by previous studies. For example, the filters used by Small and Daniloff (1967) had slopes of 35 dB/octave, and those used by Fastl were 120 dB/octave. Digital noise generation was able to reveal pitch shifts for several reasons. First, the sharp edges removed the uncertainty intrinsic to analog filtering about how the edge frequency, *f_e_*, should be defined. Second, the sharp edges led to relatively stronger pitch sensations that allowed for more precise matching. The observed pitch shifts were 5%–10% of the edge frequency, *f_e_*, in the range 200 Hz < *f_e_* < 400 Hz, and the shift percentages became smaller with increasing edge frequency. These observed shifts afford an opportunity for experimental tests of pitch perception models. Pitch shifts as reported in this paper indicate that a temporal theory, implemented here by an analytic model, is required for low edge frequencies while a place model, as envisioned by Békésy, likely applies for high edge frequencies.

### B. Plan of the paper

In Sec. II, an autocorrelation-based model of pitch is presented. The model predicts pitches based on an apparent periodicity determined by the pattern of lag times of the peaks of the autocorrelation function (ACF). Because of the unusual structure of its ACF, the noise edge stimulus is an especially powerful test for pitch models based on neural timing. A sinc function approximation to the ACF is used to predict pitches. Model predictions are compared with the data of Klein and Hartmann (1981) for both LP and HP noise. Despite its simplicity, the sinc-autocorrelation function (sinc-ACF) model successfully reproduces major features of the data. Success depends on incorporating multiple peaks of the ACF in the pitch computation. In Sec. III, a competing, place-based lateral-inhibition-based model is presented using physiological and psychophysical parameters. This place model is almost as successful in matching the 1981 data. In Sec. IV, new experimental data for LP and HP noise test the low-edge-frequency limit for edge pitch. The relative weakness of pitch in the HP case is attributed to the restricted tonotopic region for temporal coding of the low-frequency edge. In Sec. V, pitch-interval identification data show that the edge pitch qualifies as a true musical pitch. Section VI presents experimental data for LP and HP noise with high edge frequencies, testing the upper limits of edge-pitch perception. In Sec. VII, edge pitch is related to more general models and experiments. Finally, Sec. VIII is a summary. The mathematical foundation for the sinc-ACF model is given in Appendixes A and B.

## II. AUTOCORRELATION MODEL

As a consequence of phase-locking in auditory nerve fibers, the temporal pattern of neural spikes is highly correlated with the stimulus waveform, after taking into account cochlear filtering and auditory transduction. As per the models by Licklider (1959), Meddis and Hewitt (1991a,b), and Patterson *et al.* (2000), the temporal character of our model is represented by an ACF—a representation that highlights the periodic character, or approximate periodic character, of waveforms that lead to pitches. The model estimates pitches using an algorithm based on the lags of the peaks in the ACF. Noise edge pitch (NEP) offers a particularly interesting pattern of peaks.

### A. Autocorrelation and pitch

Because the noise stimuli of interest are broadband, a physiologically detailed model might begin by dividing the noise spectrum into auditory filter bands. Cochlear auditory filtering might be followed by half-wave rectification and compression, known to apply to the auditory periphery. Then, autocorrelation may be calculated within each band. Subsequently, ACFs for the separate auditory bands may be summed to generate a “summary autocorrelogram” (Meddis and Hewitt, 1991a,b) or “population interval distribution” (Cariani and Delgutte, 1996a,b). Although we have investigated models like that in the past (Cariani *et al.*, 2015; Hartmann *et al.*, 2015), the model in this report is much simpler. Specifically, it makes a linear approximation for the periphery. With that approximation, a summary autocorrelogram, summed over contiguous, rectangular bands, is mathematically the same as the ACF for the broadband stimulus as explored here. The initial modeling in this section also assumes that the noise stimulus has no intrinsic variability. The noise spectrum is approximated by its long-term average.

### B. The sinc-autocorrelation model

The long-term average power spectrum for a noise edge stimulus can be represented as a rectangle. For the LP NEP, the rectangle extends from zero to the edge frequency *f_e_*. For the HP NEP, the rectangle extends from *f_e_* to infinity. This report first treats the LP stimulus in detail and then shows how a simple modification applies to the HP condition.

#### 1. LP noise

**FIG. 1.**
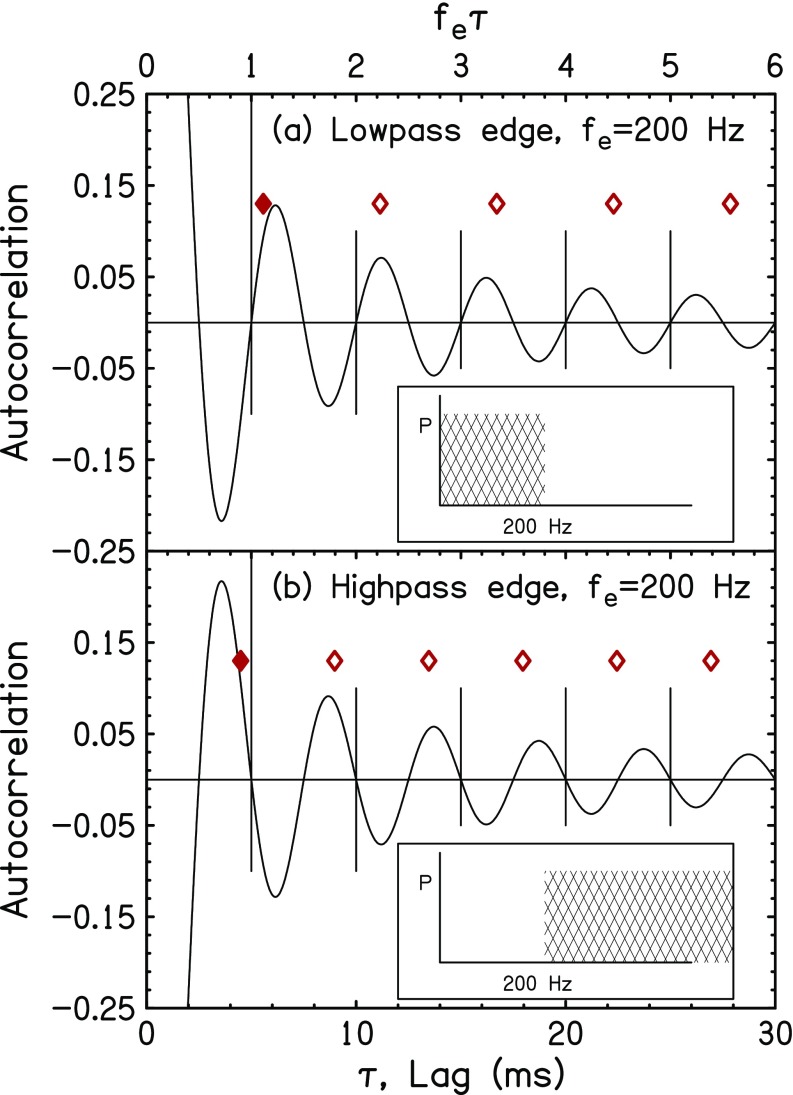
(Color online) Model sinc-ACFs for (a) LP and (b) HP noise with edge frequencies *f_e_* = 200 Hz. Vertical lines indicate periods corresponding to the edge frequency *n*/*f_e_*. The peaks of the ACFs are shifted right (LP) and left (HP) from those vertical lines leading to pitch shifts. Also, the peaks of the sinc functions are almost, but not exactly, evenly spaced. Solid diamonds show the periods $\hat{\tau }$, reciprocals of the predicted pitches [from Eqs. (3) and (4)] for a 30-ms temporal window so that the number of peaks is *N* = 5 (LP) or 6 (HP). The mathematics of Appendix A puts those two predictions between 1/*f_e_* and the first peak of the sinc-ACF at *τ*_1_ = (1 ± 1/4)/*f_e_*. Open diamond symbols show integer multiples of $\hat{\tau }$. The insets show the rectangular model power spectra.

The ACF is the inverse Fourier transform of the power spectrum. Because the long-term average power spectrum of a LP noise with mean power $\bar{P}$ is either zero or constant at $\bar{P}/(2\pi f_{e})$, the ACF is a sinc function of lag *τ*,

$$a_{LP}(\tau )=\frac{\;\text{sin}(2\pi f_{e}\tau )}{2\pi f_{e}\tau },$$ (1)

for $\bar{P}=1$. This function can be thought of as an approximation to the all-order interspike-interval histogram. The function is shown in Fig. 1 for an edge frequency of 200 Hz.

We assume that the pitch of the LP NEP is determined by the values of lag *τ* where *a*_LP_(*τ*) has peaks. The prediction of an edge pitch from the peaks in the sinc-ACF is derived in Appendix A. To a good approximation, the first peak of *a*_LP_(*τ*) (after the zeroth peak at the origin) occurs at *f_e_τ* = 5/4 cycles. To an even better approximation, all the other positive peaks are separated from the first by integer multiples of the period. Therefore, the *n*th peak occurs very near the lag value of

$$\tau_{n}=(n+1/4)/f_{e},\;n=1,2,3,\ldots ,N.$$ (2)

This approximation is tested in Appendix B.

As noted by Cariani (2004), each of these peaks is a potential temporal pitch cue, but because of the finite auditory integration time, only the first *N* of them are important to the pitch sensation. The value of *N* is a critical matter addressed in this paper. If only the first peak is included (*N* = 1), then [from Eq. (2)] the pitch cue is *τ*_1_ = 1.25/*f_e_*, and the predicted pitch becomes *p* = 1/*τ*_1_ = *f_e_*/1.25. Thus, the ratio of pitch to the edge frequency is *p*/*f_e_* = 1/1.25 = 0.8, i.e., the pitch is predicted to be 20% below the edge frequency. That prediction gets one thing right: for a LP noise, the perceived pitch is lower than the edge frequency. However, this predicted pitch shift away from the edge is too large by a factor of about 5. The experimental pitch match is usually lower than *f_e_* by much less than 20%, typically near 4%. Apparently the first peak in the ACF is an inadequate cue for the pitch shift for NEP. The problem is solved by incorporating more peaks.

The model which relates the multiple peaks of the ACF to a pitch prediction is described in Appendix A. There, it becomes evident that including more peaks (larger *N*) in the computation of pitch maintains the sign of the predicted shift but reduces its magnitude. It is reasonable to suppose that *N* is limited by a temporal window, defined by a maximum lag time *τ*_max_, over which peaks can be obtained. Because *N* is approximately equal to *f_e_τ*_max_, the number of important peaks decreases as the edge frequency decreases. It will be seen that this effect predicts that the pitch shift percentage should increase with decreasing edge frequency, if it is assumed that *τ*_max_ is relatively insensitive to edge frequency or tonotopic region. Appendix A shows that the ratio of predicted pitch to edge frequency for LP noise is given by

$$p/f_{e}=\frac{1}{1+[1/(4N)][\ln(N)+\gamma +1/(2N)]},$$ (3)

where *γ* is the Euler-Mascheroni constant, *γ* ≈ 0.57722.

**FIG. 2.**
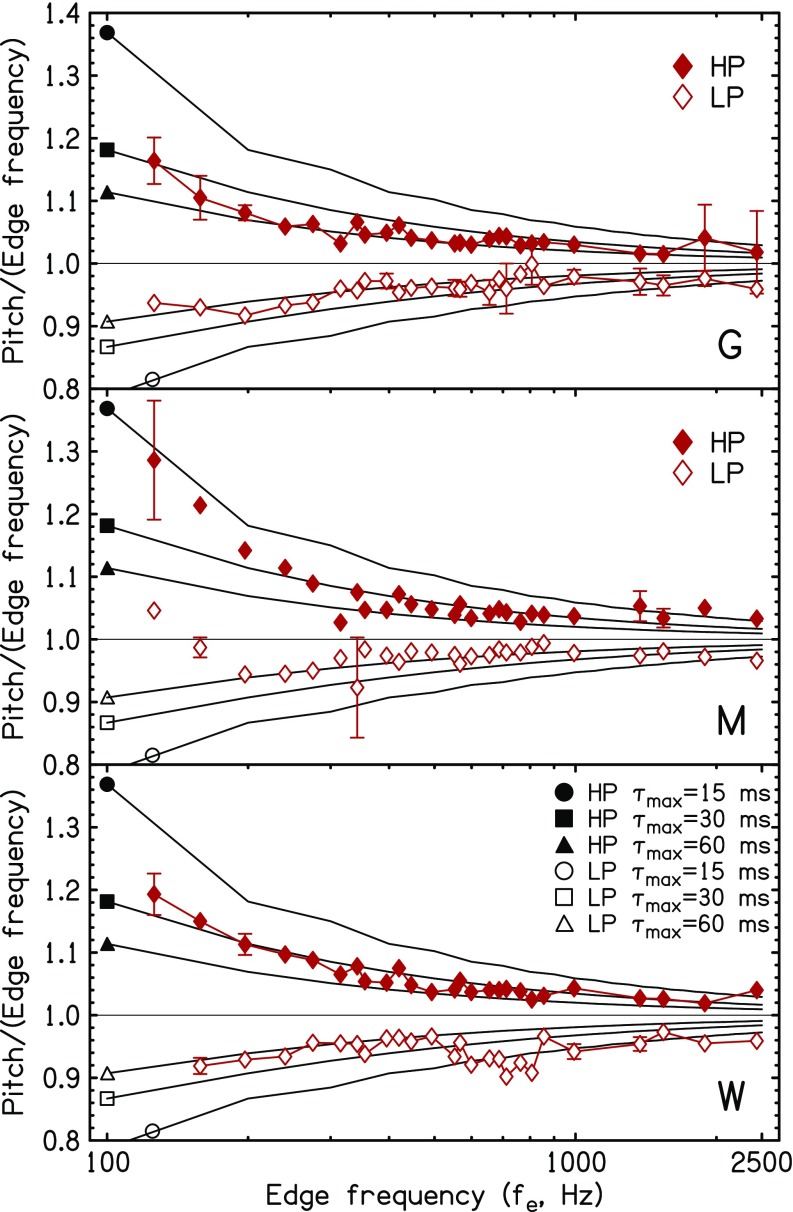
(Color online) Predicted NEPs compared to pitch matches for three listeners—*G*, *M*, and *W*. Symbols show measurements from Klein and Hartmann (1981), filled for HP noise and open for LP noise. Each diamond symbol shows the mean of at least four matches. Error bars are two standard deviations in overall length. Most error bars are smaller than the points, but error bars become larger below 200 Hz, where matching became difficult and data may be less reliable. Lines marked with circles, triangles, and squares indicate the predictions of the sinc-ACF model from Eqs. (3) and (4) for three different values of the maximum lag in the model system. The equation was evaluated only every 100 Hz. Discontinuities in slope show the effect of the INTEGER function.

This formula should hold good in the case that *N* is not too small. Also in that case, *N* ≈ *f_e_τ*_max_, and *p*/*f_e_* can be computed making that substitution for *N* in Eq. (3). However, *N* is an integer, and it is only reasonable to take the integer part of the continuous function, i.e., *N* = INT(*f_e_τ*_max_). That was assumed for the computation of the final pitch predictions shown in Fig. 2.

#### 2. HP noise

Appendix A also shows that the corresponding prediction for HP noise is

$$p/f_{e}=\frac{1}{1-[1/(4N)][\ln(N)+\gamma +1/(2N)]}.$$ (4)

Two predictions follow from the equations above: first, the pitch of HP NEP should be above the edge frequency just as the pitch of LP NEP should be below. Second, the percentage shift for HP NEP [Eq. (4)] should be somewhat larger in magnitude than the percentage shift for LP NEP [Eq. (3)]. The latter follows because:

|1/(1−x)−1|>|1/(1+x)−1|, (5)

for *x* small and positive. The temporal model introduced here for LP and HP noise bands and based on a limited number of peaks of the sinc function will be called the “sinc-ACF model.”

### C. Edge pitch listening experiment

In Fig. 2, the predictions from the sinc-ACF model are compared to the pitch matches by three listeners in the experiment by Klein and Hartmann (1981). The details of the experiment can be found in the original publication. Briefly, there were three male listeners, *G*, *M*, and *W* (ages, 22, 22, and 41 yr) with normal hearing. They used a sine tone with adjustable frequency and level to match the pitch of noise bands with sharp edges (30-dB discontinuity). The 12-bit noise stimuli were made by adding 251 equally spaced sine components—equal amplitude random phase. Stimuli were presented through headphones at 60 dBA sound pressure level (SPL) in a sound treated room.^1^

Figure 2 indicates notable qualitative agreement among the three listeners, at least above 200 Hz, where there was no exception to the rule that the mean matching frequencies were shifted into the region of spectral power for both HP and LP. The sinc-ACF model is consistent with that rule.

### D. Comparing the temporal model and experiment

Predictions from the sinc-ACF model for LP and HP NEP are shown in Fig. 2 for three values of the maximum lag, *τ*_max_ = 15, 30, and 60 ms. Figure 2 shows that the pitch shifts are larger as a percentage of the edge frequency for lower edge frequency though the experimental shifts below 200 Hz are uncertain. The model calculations agree; the predicted pitch shifts increase with decreasing edge frequency. The shifts increase because, with a given time window for autocorrelation (e.g., *τ*_max_ = 30 ms), fewer autocorrelation peaks are in a window of given duration when the edge frequency is low. Figure 2 also shows that the pitch shifts are larger in magnitude for HP edges than for LP edges. As noted in Eq. (5), this is also a feature of the sinc-ACF model.

**FIG. 3.**
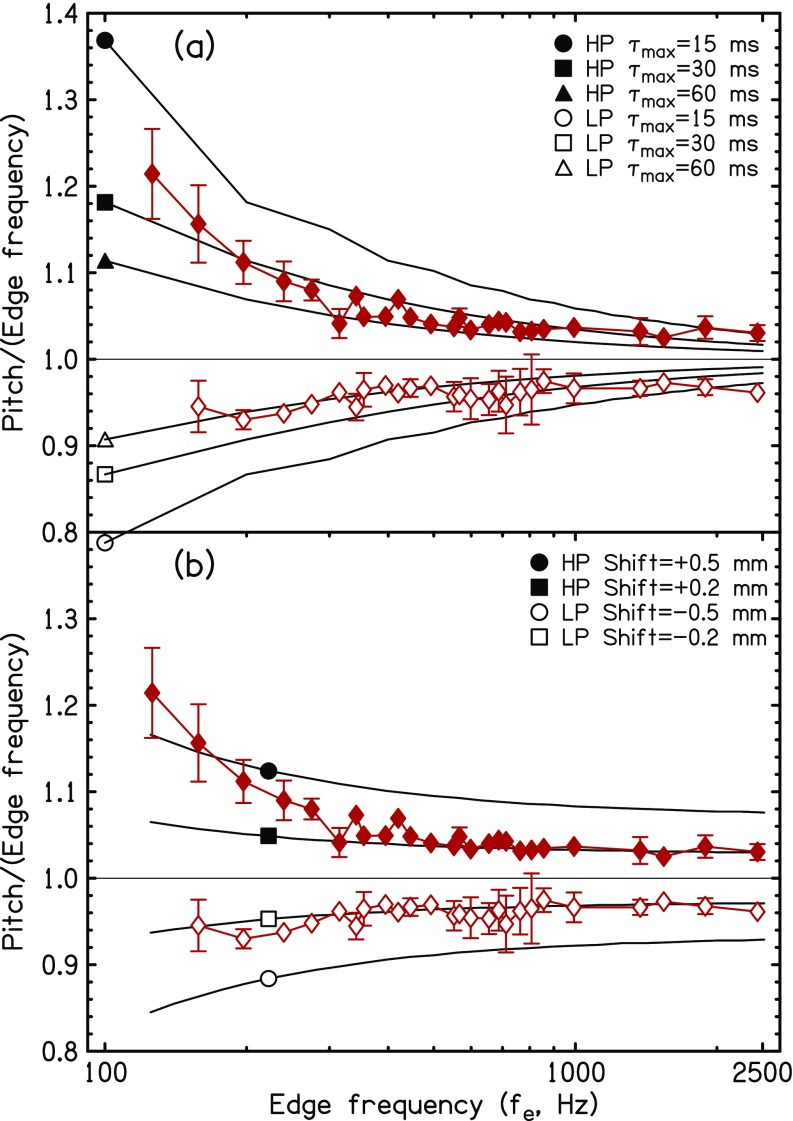
(Color online) Diamonds show pitch matching data averaged over the three plots in Fig. 2. (a) Lines marked with circles, triangles, and squares indicate the predictions of the sinc-autocorrelation model from Fig. 2. (b) Diamonds repeat the average pitch matching data from (a). Lines marked with circles and squares indicate the predictions of the lateral inhibition model with tonotopic displacement parameters for the Greenwood formula as listed in the legend.

Although the model predictions in Fig. 2 are for fixed lag windows, *τ*_max_, a careful comparison between matches and models indicates that the best fitting lag window increases in duration with decreasing edge frequency for all the listeners. The effect is evident also in Fig. 3(a) where the matching data are averaged over the three listeners. Whereas matches to edge frequencies near 2000 Hz seem to agree with the model for *τ*_max_ = 15 ms, matches to edge frequencies below about 600 Hz agree better if *τ*_max_ = 30 or 60 ms. This observation is consistent with other evidence that auditory integration times become longer as the frequency range decreases (Moore, 1982; Bernstein and Oxenham, 2005; de Cheveigne and Pressnitzer, 2006). The apparent dependence of window duration on edge frequency needs to be understood in context. Window durations depend on the characteristic frequencies of neural channels. The channels that are important for a given edge frequency *f_e_* are those with characteristic frequencies in the neighborhood of *f_e_*.

According to Eqs. (3) and (4), the predictions in Fig. 3 depend only on the maximum lag *τ*_max_ through parameter *N* ≈ *f_e_τ*_max_. We used a two-parameter model to optimize the dependence of *τ*_max_ on edge frequency to fit the data in Fig. 3(a) from 200 to 2500 Hz.

$$\tau_{\text{max}}=\tau_{2500}+(\tau_{200}-\tau_{2500})\frac{\ln(2500/f_{e})}{\ln(2500/200)}.$$ (6)

The best fitting parameters and root-mean-square (RMS) errors are given in Table I(a). It is evident that the best fit requires a longer window *τ*_200_ at low edge frequencies than at high *τ*_2500_ by a factor between 2 and 4. The window durations in Table I(a) can be compared with the window durations suggested by Moore (1982) for a pitch model based on interspike intervals (first-order histogram): a minimum duration of 0.5/*f_c_* and a maximum of 15/*f_c_*, where *f_c_* is a characteristic frequency for the tonotopic region. The maximum duration, arguably applicable to our all-order distribution, is 75 ms and 6 ms for 200 Hz and 2500 Hz, respectively. However, unlike the Moore formulas, our optimum window duration does not scale with edge frequency. The factor between 2 and 4 is much less than 12.5.

**TABLE I.** Parameter fits to edge pitch matching data. Parameter fit to the data from Figs. 2 and 3. (a) Temporal model (sinc-autocorrelation peak). (b) Place model (lateral inhibition—Greenwood). (c) Place model (lateral inhibition—Glasberg-Moore). (d) Place model (frequency-independent percentage shift). RMS error is in units of *p*/*f_e_*.

(a) Temporal			
Listener	*τ*_200_ (ms)	*τ*_2500_ (ms)	100 × RMS
*G*	60.7	23.8	1.05
*M*	50.0	20.5	1.89
*W*	44.7	10.6	1.79
All	55.6	13.9	0.99
(b) Greenwood			
Listener	Δ*z* LP (mm)	Δ*z* HP (mm)	100 × RMS
*G*	0.21	0.22	1.22
*M*	0.16	0.29	1.78
*W*	0.30	0.28	1.85
All	0.22	0.26	1.20
(c) Glasberg-Moore			
Listener	Δ*z* LP (Cam)	Δ*z* HP (Cam)	100 × RMS
*G*	0.25	0.26	1.15
*M*	0.19	0.35	1.69
*W*	0.35	0.33	1.82
All	0.26	0.31	1.10
(d) Constant			
Listener	Δ*f* LP (%)	Δ*f* HP (%)	100 × RMS
*G*	3.8	4.0	1.62
*M*	2.9	5.4	2.21
*W*	5.5	5.0	2.13
All	4.1	4.8	1.67

As evident in Eq. (1), the ACF oscillates with an approximate spacing given by the reciprocal of the edge frequency. Therefore, a pitch model that identifies pitch perception with the *spacing* of the oscillations of the ACF must fail in view of the observed pitch shifts. Reasoning like that caused Klein and Hartmann (1981) to abandon temporal models for edge pitch. By contrast, the autocorrelation model of Secs. II A and II B above uses the actual lag values of the peaks and not their regular spacing. Similarly, Yost *et al.* (1996, 1998) and Patterson *et al.* (2000) accounted for the pitch of iterated rippled noise in terms of the lag value of the first peak of the ACF. However, modeling NEP requires more than just the first peak because the first peak alone predicts a pitch shift that is too large.

## III. LATERAL INHIBITION PLACE MODELS

Lateral inhibition is a neural phenomenon known to occur in the visual system (Hartline *et al.*, 1956). Lateral inhibition has been a hypothetical element in model auditory systems (Békésy, 1963), and masking data have been interpreted in terms of it (Carterette *et al.*, 1970a,b). The lateral inhibition concept can account for NEPs in a very natural way because it enhances contrast at edges. Plausible quantitative models can be expected to predict the pitch shifts into the noise, as observed experimentally, because lateral inhibition causes the peaks to be on the large-excitation side of the edge. The purpose of this section is to examine the predictions of plausible lateral inhibition models and compare the predicted pitch shifts with observed shifts for NEP.

Lateral inhibition models are tonotopic, ultimately related back to the displacement pattern on the basilar membrane.^2^ Because of the approximately logarithmic nature of the human tonotopic axis, it is natural to choose shifts of the excitation pattern on a logarithmic scale. For example, Shamma (1985) used 1/3 octave. Such a shift would lead to a flat line prediction for *p*/*f_e_* on a logarithmic plot like Fig. 3(b). It would not capture the tendency for shifts to increase as edge frequency decreases.

The model considered here relates frequency to basilar membrane place through the Greenwood formula (Greenwood, 1961),

$$f(z)=165(e^{az}-1),$$ (7)

where *f* is the frequency in Hertz and *z* is the tonotopic coordinate in mm measured from the apex. Parameter *a* is 0.14 mm^−1^ for human cochleas. The Greenwood formula, based on psychoacoustical masking experiments, shows a low-frequency compression of the tonotopic coordinate when plotted as a function of the logarithm of the best frequency, i.e., changing the best frequency by a semitone corresponds to a smaller change in tonotopic coordinate (Δ*z*) at low frequency than at high. Such low-frequency compression of the tonotopic scale is characteristic of auditory filter models of the periphery, e.g., Zwicker (Bark scale; 1961), Glasberg and Moore (Cam scale; 1990).

Our procedure for computing the peak caused by lateral inhibition first uses the Greenwood formula to convert the edge frequency to a tonotopic place *z*. Then it calculates the place of peak excitation z′ by applying a constant shift in millimeters, and finally it reconverts place z′ to a frequency to represent the pitch *p*. In order to model edge enhancement, the peak shift must be positive for HP edges and negative for LP edges. The comparison is shown in Fig. 3 where the data to be modeled are the same in Figs. 3(a) and 3(b). Figure 3(b) shows that the shift calculated from the lateral inhibition model has the right sign and approximately the right shape to agree with the data. Because of the low-frequency compression of the tonotopic scale, the magnitude of the predicted shift is larger for lower frequencies than for high, in agreement with pitch matching data. However, the compression is only modest, and the curvature of the predicted shift functions is smaller for the lateral inhibition model [Fig. 3(b)] than for the temporal model [Fig. 3(a)].

The lateral inhibition model was tested quantitatively against the data in Figs. 2 and 3 in the frequency range from 200 to 2500 Hz (actually 197 Hz to 2438 Hz). Tables I(a) and I(b) show the results of parametric best fits to the experimental matches. For the temporal model [Table I(a)] the fitting parameters are low- and high-frequency window durations in milliseconds. For the Greenwood place model [Table I(b)] the fitting parameters are peak displacements in millimeters. Overall, the RMS fitting errors are somewhat smaller for the temporal model, and the cause of this difference is the relatively flat nature of the predictions by the place model.

The rectangular bandwidth scale by Glasberg and Moore (1990) becomes an alternative tonotopic scale if the bands are stacked in order of center frequency. The formula is the same as the Greenwood formula, but the parameters are different,

$$f(z)=229(e^{az}-1),$$ (8)

where *f* is the frequency in Hertz and *z* is the auditory filter number in Cam units, numbering the Glasberg and Moore equivalent rectangular bands. Parameter *a* is 0.108 Cam^−1^ based on notched noise masking. Table I(c) shows that the place model using the Glasberg-Moore formula [Eq. (8)] fits the edge pitch data somewhat better than using the Greenwood formula [Eq. (7)], but the fit is still slightly inferior to the temporal model.

Finally, the frequency-dependent predictions shown in Figs. 3(a) and 3(b) were replaced by the best straight lines—separately for LP and HP conditions. The straight lines correspond to a temporal model in which the duration of the lag window (*τ*_max_) depends on frequency range in such a way that the number of autocorrelation peaks (*N*) within the window is always the same, independent of edge frequency. Therefore, *τ*_max_ ∝ 1/*f_e_*. The straight lines also correspond to a place model in which the displacement of the excitation peak caused by lateral inhibition is a constant fraction of the edge frequency as in the appendix in Shamma (1985). Table I(d) shows that RMS errors are largest for these straight line fits indicating that the frequency dependences introduced into the temporal and place models make useful (though small) contributions.

All of the models compared in Table I have two adjustable parameters, which makes the comparisons fair. However, the place models from Tables I(b) and I(c) both require parameters to relate place to frequency, determined originally from other experiments. The temporal model is more economical in that respect. The sign of the pitch shift results from the logic of lateral inhibition and the fitting procedure for the place model, but it emerges automatically from the temporal model.

## IV. LOW-EDGE-FREQUENCY EXPERIMENTS

Small and Daniloff (1967) reported that listeners were unable to do octave matching for HP noise with edge frequencies less than 610 Hz, but octave matches could be made for LP noise with an edge as low as 145 Hz. Similarly, Fastl (1971) found that highpassed noise with an edge frequency below 500 Hz did not produce a pitch. Fastl and Stoll (1979) asked listeners to rate the pitch strength of 12 different stimulus types, including LP and HP noises with edges ( ±192 dB/octave) at 125, 250, and 500 Hz. Their listeners found LP edge pitches to be stronger and HP edge pitches to be inaudible sometimes. Also, the data from Klein and Hartmann tended to show more variability for HP noise than for LP noise as the edge frequency decreased. By contrast, with window durations as long as 60 ms, the sinc-ACF model does not immediately suggest any particular difficulty for the HP condition. Low-edge-frequency experiments were done using our sharp edges to test the low-edge-frequency limit and compare with the previous experiments. The limit for HP noise was of particular interest.

### A. HP noise

#### 1. Procedure

The HP noise bands were computed in the frequency domain with a sharp edge at *f_e_* at the low-frequency end, where the spectral discontinuity, as presented to the listeners, was more than 40 dB. Above *f_e_* the noise was equal-amplitude, random-phase extending to 20 000 Hz except that the amplitude decreased linearly by 20 dB between 16 000 and 20 000 Hz to avoid a sharp edge at very high frequency. Because noise bands were very wide for all edge frequencies, the noise power was essentially constant. Noise bands were generated with 16-bit precision at a sample rate of 100 000 samples per second. They were presented diotically to listeners in a double-walled sound room through Sennheiser HD600 headphones (Wedemark, Germany). The level was 65 dBA. Noise stimuli were 520 ms in duration with 20-ms raised-cosine onsets and offsets.

A noise interval was followed by a 400-ms silent interval^3^ and then by a 500-ms sine tone with a frequency that could be adjusted by the listener to match the pitch of the noise edge. The entire audible range of frequencies was available for the matching tone through a combination of push-button range switching and a ten-turn potentiometer on the response box. The control voltage from the response box was read by a 12-bit analog-to-digital converter, and the matching tone was then generated digitally so that the matching frequency was known precisely. The relationship between the potentiometer setting and the tone frequency was randomly offset at the start of each trial. The level of the matching tone was also adjustable by the listener, and the matching tone could be muted with the press of a button. The cycle of target noise and matching tone repeated indefinitely until the listener was satisfied with the match. After making a match, the listener received feedback, including the matching frequency, the edge frequency, and the percentage difference between the two. Listeners were told to expect matches to be close to the edge, but not necessarily identical to the edge. (See footnote 8 in Sec. VIII.) Our experiment looked for pitches far below the limits found by Fastl (1971) and Small and Daniloff (1967). It used eight edge frequencies: 50, 70, 100, 150, 200, 280, 400, and 560 Hz. The different edge frequencies were presented in random order in an experiment run.

As a control experiment, listeners adjusted the frequencies of sine tones to match the pitches of eight low-frequency sine tones, ranging from 40 to 500 Hz. These were presented at a nominal level of 70 dB SPL, considering the elevated audiogram at 40 Hz.

#### 2. Listeners

There were six male listeners in the matching experiments overall. Listeners *A*, *B*, *I*, *S*, and *Z* were between the ages of 20 and 25 yr. They were accepted as listeners based on their ability in a high-frequency sine-sine pitch matching test. Listeners *A*, *I*, and *S* were able to match sine tones with a standard deviation of less than 10 cents (0.6%) at least up to 13 kHz. Listener *B*'s standard deviation was less than 10 cents between 350 and 9000 Hz. Listener *Z* was tested only up to 8 kHz where his standard deviation was 10 cents. Listener *W*′ was the same as Listener *W* (data in Fig. 2) but tested 37 yr later. He only participated in low-frequency experiments. All listeners were amateur musicians except for listener *A*, who was a professional. The young listeners were all students at Michigan State University; they signed a consent form approved by the University Institutional Review Board (IRB).

#### 3. Results

**FIG. 4.**
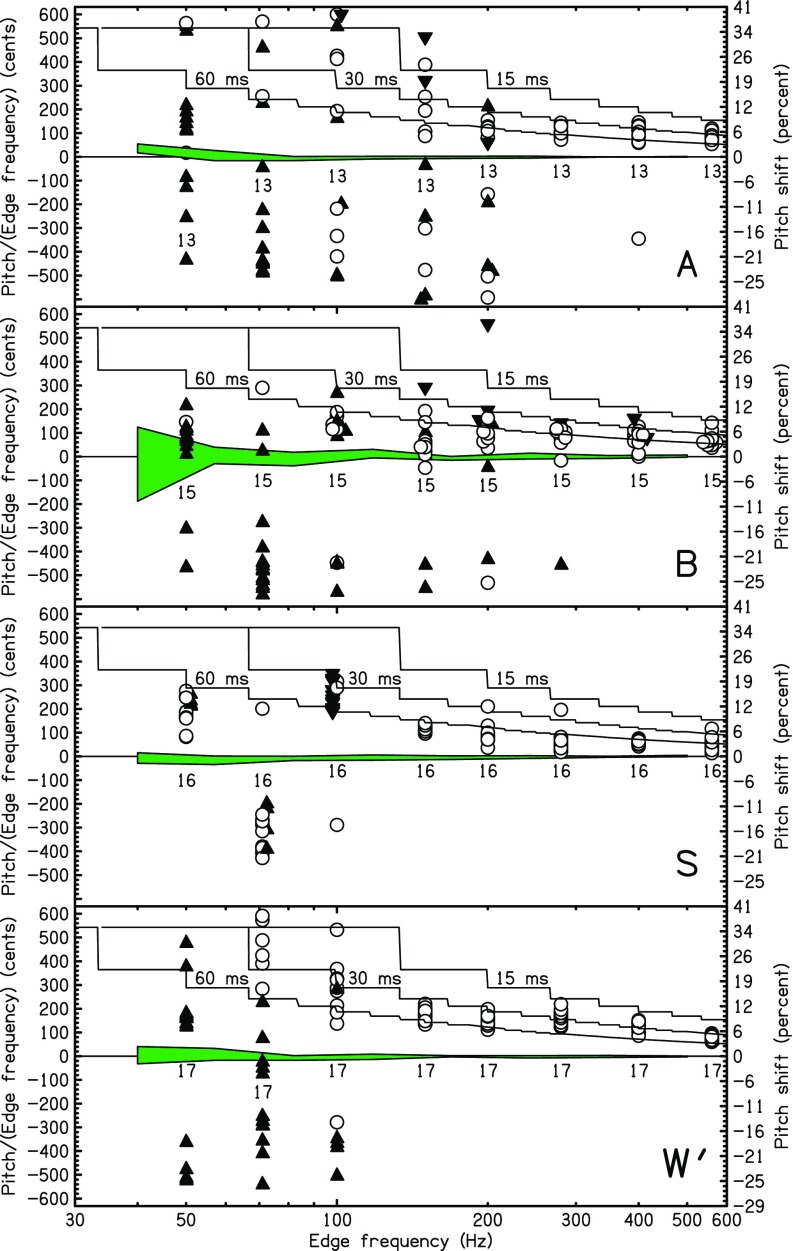
(Color online) Probing the low-frequency limit with HP noise. The ratios of matching frequency to edge frequency are expressed in cents and shown by circles. Matches that were an octave (occasionally two octaves) higher (lower) than plotted here are shown by an upward (downward) triangle. Small numbers indicate the number of matches (trials) for each frequency. The shaded region near zero cents is centered on the average match (six trials) for sine tones in the control experiment, and the width of the region is two standard deviations. Solid lines give the prediction of the sinc-autocorrelation model for temporal windows of 15, 30, and 60 ms. At these low frequencies there are few sinc function peaks in the temporal window, and the predicted plots show a succession of plateaus as new peaks enter the window. The plateaus appear because the plots use a fine mesh of points.

Four of the listeners participated in the low-edge-frequency HP experiments. The matching data are shown in Fig. 4. Small numbers near the horizontal axis show the total number of trials. Matching ratios are shown by circles. Matching ratios one octave (occasionally, but rarely, two octaves) higher than plotted are shown by upward pointing, filled triangles. For instance, an upward triangle at *f_e_* = 70 Hz plotted near −300 cents represents a matching tone that was about 900 cents above 70 Hz (900–1200 = −300). Downward pointing triangles indicate matches that were an octave lower than plotted. Solid lines show the predictions of the sinc-autocorrelation peak model for different window durations. The model is the same as for Figs. 2 and 3, but the plots look different because the model was evaluated at a fine mesh of points for Fig. 4.

Comparison with the predictions confirms the observation made in Sec. II that the optimum window duration is relatively long for edge frequencies below 600 Hz. Most of the matching frequencies in Fig. 4 agree best with the model with a 60-ms window.

All listeners made consistent matches for edge frequencies above 200 Hz with negligible octave errors and most of the matches within a semitone of the target. Listeners *S* and *W*′ matched consistently down to 150 Hz. Consistent matches were almost all above the edge frequency as expected from the sinc-autocorrelation peak model. Listeners found the pitches evoked by those edges to be salient. By contrast, for edge frequencies below 150 Hz, there was no evidence for salient pitches. There were frequent octave errors, and many matches were well away from the target. Matches showed no consistent pattern except possibly for listener *S*. However, the consistency seen for listener *S* does not seem to indicate actual edge pitch perception below 150 Hz.^4^ We conclude that the low-frequency limit for highpassed edge pitch is between 100 and 150 Hz, though values that low were not achieved by all listeners.

### B. LP noise

**FIG. 5.**
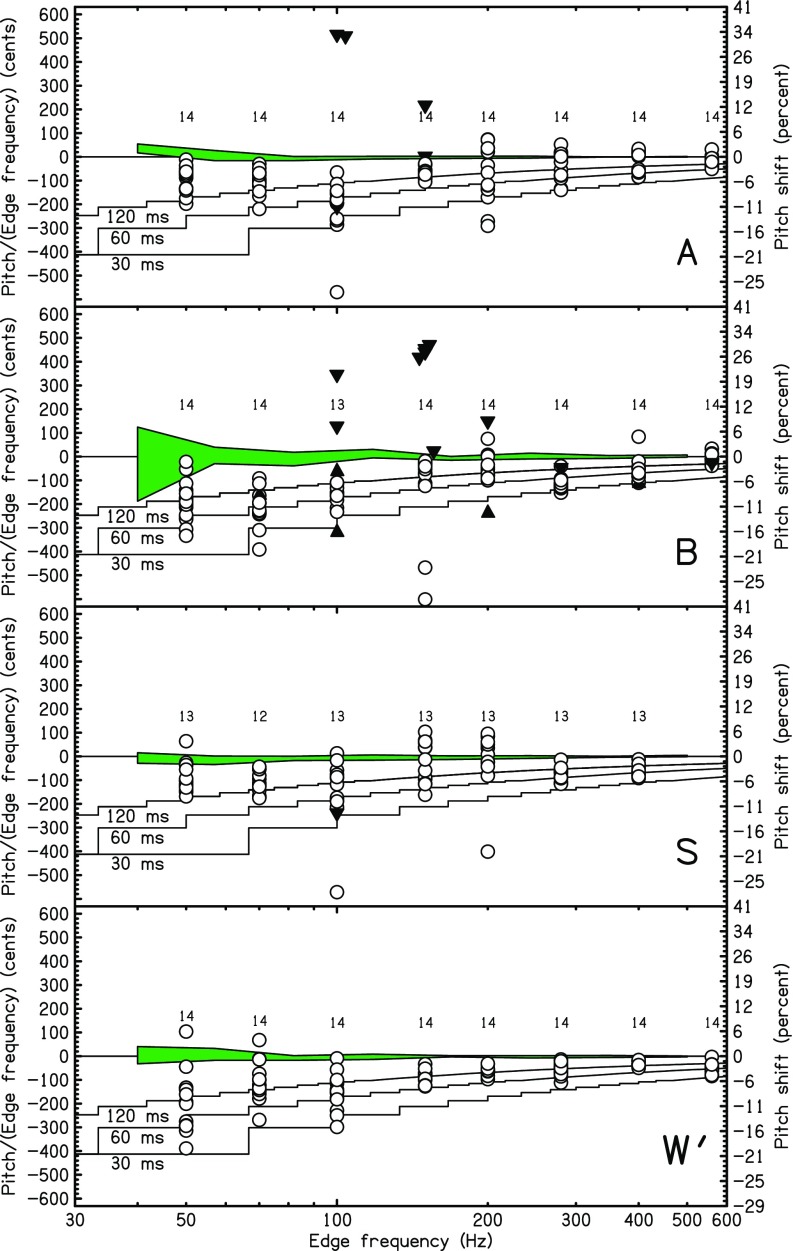
(Color online) Same as Fig. 4 except that the stimulus was LP noise.

Low-edge-frequency experiments were done using LP noise. The same edge frequencies and protocol from the HP experiments of Sec. IV A above were used. The levels were the same, too, except that the listener had the option of requesting a level increase for the lowest edge frequencies, particularly 50 Hz. Again, listeners *A*, *B*, *S*, and *W*′ participated, and results are shown in Fig. 5. The data show that matches could be made reliably down to 50 Hz, the lowest edge frequency tested. Although the matching variance grew for decreasing edge frequency, the growth may relate more to low-frequency loudness and pitch acuity, in general, than to the strength of edge pitch. As expected for lowpassed noise, most (89%) of the pitch shifts were negative, though the percentage fell to 81% for listener *A*.

### C. Discussion

A comparison of the HP and LP experiments shows that the LP edge pitch could be heard for edge frequencies at least an octave lower than the limit for the HP edge pitch. The difference can be understood within a temporal model by considering the amount of tonotopic axis available to represent the timing for these two noise types. The difference is inconsistent with a place model that incorporates auditory filters with the usual tuning asymmetry.

#### 1. Temporal model

For LP noise with a low edge frequency, the entire tonotopic axis with best frequencies (BF) above the edge experiences no on-frequency excitation. Instead, neurons with high BF mainly experience the excitation that is near the edge, especially because their low-frequency slopes are relatively shallow. Calculations with a gammatone filter model show an ACF with peaks at lag values determined by the edge frequency, independent of BF when BF is greater than the edge frequency. A strong ACF over a major part of the tonotopic axis can be expected to lead to a strong pitch, in agreement with experiment. At the same time, a LP noise with a low edge frequency has relatively few components in its spectrum, and that leads to a rough-sounding temporal envelope making the matching experience unpleasant and somewhat difficult.

For HP noise with a low edge frequency, the only neurons free of on-frequency excitation are in the region of the tonotopic axis with even lower BF. Because of their sharp cutoff in the high-frequency tails, these neurons experience only little excitation having a temporal structure determined by the edge frequency. Therefore, HP edge pitch can be expected to disappear for low edge frequency, as observed experimentally.

The above explanation for the differences between LP and HP noise for low edge frequency retains the temporal character of our autocorrelation model but augments it with place considerations to obtain a qualitative understanding of the strength of the temporal information that is available. Because excitation pattern models are well developed, it would be possible to make quantitative predictions for the relative strengths of edge pitches with different edge frequencies. Such calculations are beyond the scope of the present paper.

#### 2. Place model

For LP noise with low edge frequency, the edge of the excitation pattern is broad because neurons with BF near, and slightly above, the edge are excited by noise components that are below the neuron BF. For HP noise with low edge frequency the edge of the excitation pattern is sharp because neurons with BF just below the edge are inefficiently excited by noise above their BF. Therefore, edge pitch is predicted to be stronger for HP noise, contrary to experiment.

## V. INTERVAL IDENTIFICATION

Musical pitch is recognized if a listener can identify melodies without rhythm or adjust or identify musical intervals (Houtsma and Goldstein, 1972; Plack and Oxenham, 2005; Moore and Ernst, 2012; Oxenham *et al.*, 2011; Gockel and Carlyon, 2016). To determine whether NEP qualifies as a musical pitch, interval identification experiments were inserted into the schedule of pitch matching runs for high and low edge frequency. Listeners made open set identifications of melodic intervals to verify that the pitch elicited by noise bands with a sharp edge qualifies as a musical pitch. Because the musical nature of the edge pitch itself was in question, there was no special concern with frequency range, and edge frequencies ranged from 600 to 2400 Hz.

### A. Intervals—LP noise

In the LP experiment, the intervals and noise edge frequencies (Hz) were these: octave (600,1200), fifth (1600,2400), fourth (1200,1600), and major third (1600,2000). These four intervals were always melodic and ascending. For each experimental trial, an interval was randomly chosen from the four and presented to the listener four times—a standard cycle. After the cycle the listener could either identify the interval or could request a repetition of the cycle. Listeners were familiar with musical intervals, but they did not know which intervals were in the test. Stimuli were generated according to the procedure described in Sec. IV A. Stimuli were again 520 ms in duration with 20-ms raised cosine onsets and offsets. A pause of 400 ms separated the two noises of an interval.

Results of the experiment were as follows:

• Listener A immediately identified all four intervals without waiting for the four intervals of a cycle to complete.
• Listener *I* correctly identified the major third and the fourth after one cycle. He required two cycles to correctly identify the fifth, and misidentified the octave as a perfect fifth.
• Listener *S* correctly identified three intervals but called the major third a minor third. When then presented with a minor third (2000,2400 Hz), the listener responded, “major third.” Upon further testing with major thirds (3200,4000) and (3600,4500) and a minor third (3000,3600), the listener made one error, calling (3200,4000) a minor third.
• Listener *W*′ correctly identified all four intervals but required two cycles to identify the major third and the fourth. Listener *W*′ designed the experiment and knew which four intervals were in the set, but not the order of presentation.
• Listener *Z* correctly identified all four intervals after hearing one standard cycle for each.

### B. Intervals—HP noise

In the HP experiment, the intervals and edge frequencies were the same as those in the LP experiment. Another interval, a minor third, (2000,2400) was added to the standard set.

• Listener A correctly identified four intervals, usually before the completion of a cycle, but he misidentified the octave, insisting that it was a minor 7th! Such a misidentification might have been predicted. The octave interval was made with relatively low edge frequencies, 600 and 1200 Hz, where the pitch shift gradient is large and negative (Fig. 2). The pitch of the 600-Hz edge is expected to be increased more than the pitch of the 1200-Hz edge, leading to a compression of the perceived interval.^5^
• Listener *S* correctly identified all five intervals, always at the end of a single standard cycle. He made no mistakes in seven random trials.

### C. Discussion—Interval identification

The results of the LP and HP interval identification experiments, as summarized in Table II, indicate that noises with a sharp spectral edge elicit a musical pitch. Although some listeners made some mistakes and others required more than a single cycle, the difficulties appear to represent only isolated cases with possible additional confusion from the pitch shifts. The edge-pitch noise stimuli are clearly capable of generating a musical pitch. Isochronous melodies made with edge pitches have been recognized by audiences at conferences (e.g., Hartmann *et al.*, 2015). This positive result is hardly surprising. Akeroyd *et al.* (2001) found that binaural analogs of monaural edge pitches lead to musical pitch sensations, and binaural edge pitches are more challenging to listeners than the monaural NEPs investigated here.

**TABLE II.** Summary of interval identification performance. Fraction of intervals correctly identified.

LP					
Listener	*A*	*I*	*S*	*W*	*Z*
Fraction correct	4/4	3/4	3/4	4/4	4/4
Cycles attended	<1	2	1	2	1
HP					
Listener	*A*		*S*		
Fraction correct	4/5		5/5		
Cycles attended	<1		1		

## VI. HIGH-EDGE-FREQUENCY EXPERIMENTS

If the pitch of a noise band with a sharp spectral edge is the result of a temporal process, as conjectured in Sec. II, then the pitch sensation requires the synchrony of neural firing. Neural synchrony, at all levels of the auditory system, is known to decrease dramatically with increasing frequency, though the frequency at which synchrony is no longer operative is a subject of ongoing debate. Whereas frequency difference limen data from Moore and Ernst (2012) suggested a limit from 8 to 10 kHz, scaling arguments (Joris and Verschooten, 2013) and cochlear measurements (Verschooten *et al.*, 2018) suggest a limit no higher than a few kilohertz. At the level of the auditory nerve, it is common to set an upper limit near 5000 Hz based on Johnson's data on cat (Johnson, 1980). To determine whether edge pitch persists at high frequencies, we performed pitch matching tests for LP and HP noise with high edge frequencies. As a control experiment, we performed similar tests with sine tone targets.

### A. LP matches

**FIG. 6.**
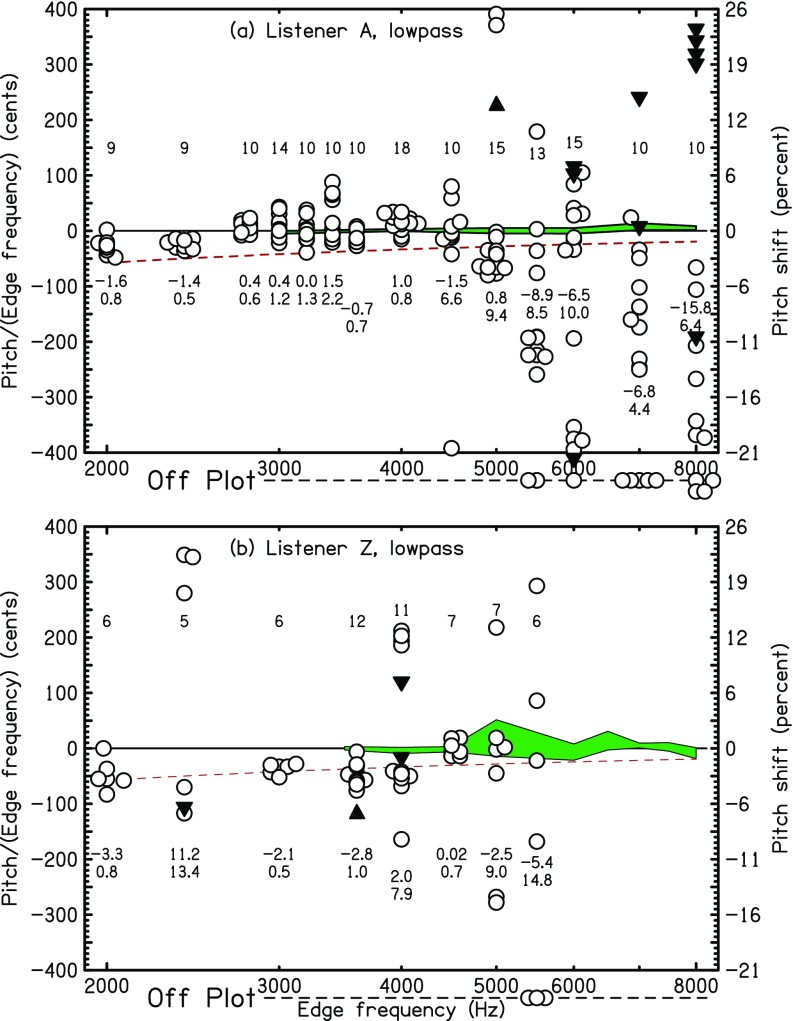
(Color online) The ratios of matching frequency to edge frequency for listeners *A* and *Z* with LP noise are shown by circles. Some are slightly displaced horizontally for clarity. Matches that were an octave higher than plotted here are shown by an upward triangle. Matches that were an octave lower than plotted here are shown by a downward triangle. Small numbers in the lower rows show the mean and standard deviation of the percentage shift. Small numbers in the upper row indicate the number of matches (trials) included in the average for each frequency. Matches with discrepancies greater than ±400 cents are plotted above/below the main graph. The dashed line is the prediction of the sinc-autocorrelation model for a lag window of 15 ms. A longer window, such as 30 ms, would fit better for listener *A*. The shaded region near zero cents is centered on the matches for sine tones, and the width of the region is two standard deviations.

**FIG. 7.**
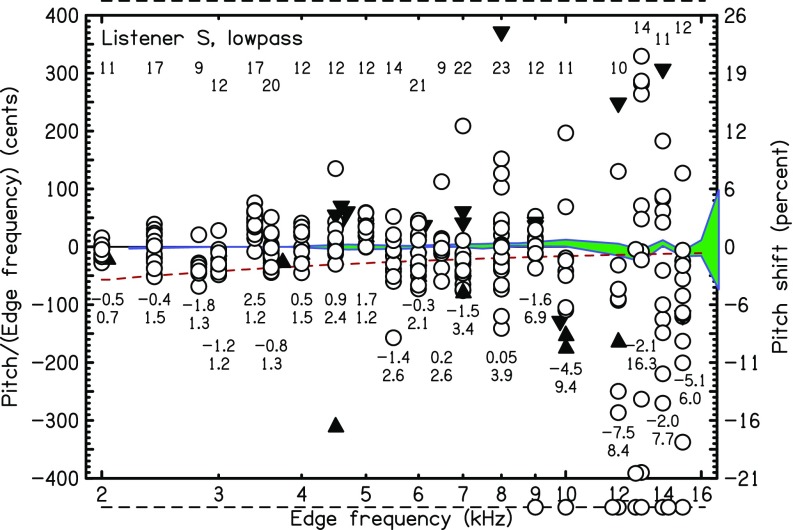
(Color online) Same as Fig. 6 but for listener *S*.

Pitch matches to LP noises are shown in two ways in Figs. 6 and 7: (1) The circles show the ratios of matching frequencies to edge frequencies, on a scale of cents, when the ratios were in the range −400 to 400 cents. Errors identified as octave discrepancies are shown by filled triangles. An upward pointing triangle indicates a match one octave above the plotted symbol. A downward pointing triangle indicates a match one octave (occasionally two octaves) below the plotted symbol. Some matches did not fall on the plot, even allowing for octave discrepancies. These are shown by circles on dashed lines above (match too high) and below (match too low) the plot. (2) The average value and standard deviation of the difference between the matching frequency and the edge frequency is shown as a percentage of the edge frequency. Matches with octave discrepancy assignment were not included in the averaging. Matches in the one-octave range from −600 cents to +600 cents discrepancy were included in the average. The numbers of matches included in the average are shown by small numbers in the upper half plane. The averages themselves, followed by the standard deviation, are in the lower half plane. The shaded region indicates the matches in a control experiment where the listener matched a sine tone target with a sine tone probe. The center of the shaded region indicates the mean and width of the region is two standard deviations in overall width.

Figure 6 shows that for listeners *A* and *Z*, matches became highly unstable as the edge frequency approached 5000 Hz. The same was true for listener *I*, but his data are not shown because his matches at 2000 Hz were not consistent enough to make a strong contrast with matches at higher edge frequencies such as 5000 Hz. For listener *A*, the standard deviation was less than a semitone for the eight edges below 4.5 kHz and greater than a semitone for the six highest edge frequencies—4.5 kHz and above. For listener *Z*, the standard deviation was greater than a semitone for the two edges above 4.5 kHz.

Listener *S* (Fig. 7) was an exception. Experiments with listener *S* began in the same range as for the other listeners, 2–8 kHz. With time, it became evident that this listener could make successful matches for edge frequencies well above 5 kHz.^6^ Therefore, listener *S* was retained for another four months and experiments were restarted using the range from 2 to 16 kHz. Figure 7 shows that the matching standard deviation was less than a semitone for the 14 edges below 9 kHz and was greater than or equal to a semitone for the 6 edges at 9 kHz and above.

### B. HP matches

**FIG. 8.**
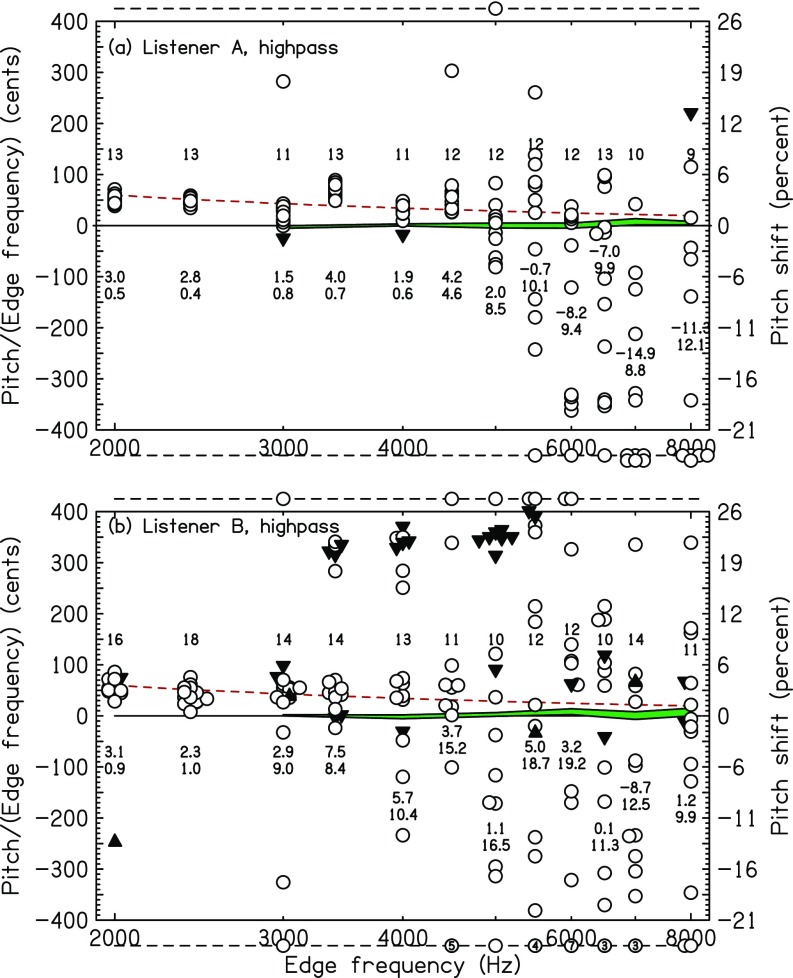
(Color online) High-frequency HP matches for listeners *A* and *B*. The dashed line is the HP prediction from the sinc-autocorrelation model for a lag window of 15 ms. Other plot features are similar to Fig. 6.

The pitch matches by listeners *A* and *B* for HP noise are shown Fig. 8. Similar to the matches for LP noise for listeners *A* and *Z* (Fig. 6), the scatter among the matches increases near 5000 Hz. For listener *A*, the standard deviation was less than one semitone for the six edges below 5 kHz and greater than one semitone for the six edges at 5 kHz and above. For listener *B*, the standard deviation was less than one semitone for the two edges below 3 kHz and greater than one semitone for the ten edges at 3 kHz and above.

**FIG. 9.**
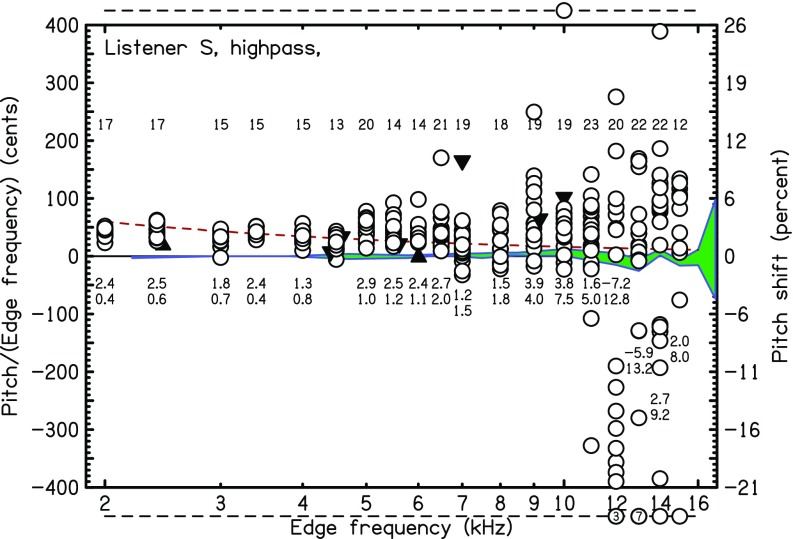
(Color online) High-frequency HP matches for listener *S*.

The exceptional listener, listener *S* was extensively tested with HP noise with high-frequency edges. His results are shown in Fig. 9. Listener *S* is indeed extraordinary. First, in the control experiment with sine tone matching, his standard deviation for 16-kHz tones was less than 15 cents. Only at 17 kHz did his standard deviation (86 cents) approach the 105 cents difference between 16 and 17 kHz. For edge pitches, the standard deviation was less than one semitone for the 13 edges below 10 kHz and greater than 1 semitone for 5 of the 6 edges at or above 10 kHz. It appears that a 12-kHz edge was not distinguishable from a 10-kHz edge.

It is very unlikely that the extraordinary performance of listener *S* was the result of an experimental artifact. The noise bands with spectral edges and the matching sine tones were generated by different electronic systems. It is hard to see how a stimulus generation artifact common to the noise and tone could serve as a cue. Further, the matching ability for very high frequencies was resistant to the introduction of LP masking noise (100–2000 Hz, 50 dBA). Experiment runs, including very high-frequency edges, always also included medium-frequency edges (2–4 kHz). Therefore, the listener was required to make large leaps in pitch range throughout.

It seemed possible that the matches by listener *S* were facilitated by the procedure whereby experimental runs either presented all HP noises or presented all LP noises. To test this idea, listener *S* did 26 runs where each run had 4 HP and 4 LP noises randomly ordered. Edge frequencies ranged from 400 to 12 000 Hz. The results showed that high-frequency edge pitch matching accuracy was not adversely affected by this mixing procedure. Standard deviations for edges at 6, 7, and 9 kHz were less than 1%. The standard deviation at 10 kHz was only 1.2%, but the standard deviation at 12 kHz was again large.

Clearly, the data from listener *S* do not agree with a model which posits a temporal origin for edge pitch with consequent limitation to 5 kHz. There are several possible explanations: perhaps listener *S* has an auditory system that preserves neural timing up to frequencies that are an octave higher than other humans—also cats (Johnson, 1980). Alternatively, listener *S* found some other process, presumably based on place mechanisms, for matching pitches. Unlike other listeners, listener *S* may be responsive to a tonotopic excitation pattern sharpened by an abrupt disappearance of inhibition at a high-frequency edge.

### C. LP and HP NEP

Two of the listeners in the high-frequency experiments (listeners *A* and *S*) participated in both the LP and the HP versions testing the high-frequency limit for NEP. Identical edge frequencies were used for both versions enabling a paired perceptual comparison between LP and HP NEP. The relevant data are the standard deviations appearing in Figs. 6(a), 7, 8(a), and 9 for these listeners. For listener *A*, the standard deviation was smaller for HP NEP than for LP NEP for 8 out of 11 edge frequencies, and the 3 exceptions were all for *f_e_* > 5000 Hz, where matching was difficult for listener *A*. Similarly, for listener *S*, the standard deviation was smaller for HP NEP than for LP NEP for 15 out of 18 edge frequencies, and the 3 exceptions were all for *f_e_* > 11 000 Hz. Listener *S* remarked informally that he found HP NEP easier to hear, even though HP NEP was presented with low-frequency masking noise.

#### 1. Temporal model

A straightforward explanation for the advantage of HP noise over LP noise for high *f_e_* is the flip-side of the explanation of the advantage of LP noise for low *f_e_*. A HP noise with an edge frequency of 2 kHz or above leaves much of the pitch-critical apical region of the cochlea unexcited by low-frequency components. Further, calculations with a gammatone filter bank show that this region is excited by the remote high-frequency components. The size of the ACF oscillations decreases as the best frequency becomes ever smaller than the edge frequency but, because it is normalized, the ACF does not disappear and it retains the periodicity expected from the sinc-autocorrelation model for BF at least several octaves below the edge frequency. For a LP noise with high edge frequency the (basilar) region for spectrally remote excitation is smaller. Therefore, a temporal model predicts that HP noise leads to a stronger pitch sensation, in agreement with experiment.

#### 2. Place model

For LP noise with high edge frequency, the edge of the excitation pattern is broad because neurons with BF near the edge are excited by noise below their BF. For HP noise with high edge frequency, the edge of the excitation pattern is sharp because neurons with BF near the edge are excited by noise above their BF. Therefore, the usual asymmetry of auditory tuning predicts that edge pitch should be stronger for HP noise, again in agreement with experiment. For very high edge frequency, the place model has an advantage over the temporal model because it does not require neural synchrony.

## VII. DISCUSSION

The edge pitch stimuli are of special interest for temporal models of pitch because of the pattern of peaks in their ACFs. The pattern can be viewed with reference to a *periodic* stimulus for which the ACF exhibits a set of regularly occurring major and minor peaks. The major peaks are found at time delays (lags) corresponding to integer multiples of the fundamental period (*n* = 0,1,2,…). Patterns of minor peaks in the ACF depend on the amplitudes of harmonics in the stimulus. By contrast, the peaks of the ACF for aperiodic noise with a sharp spectral edge are displaced from integer multiples by 1/4 unit. Therefore, the edge pitch stimuli represent a temporal analog to the “pitch shift” stimuli (e.g., Schouten *et al.*, 1962) in that the predicted pitch appears as a best fitting parameter in a model that is ideal for a periodic stimulus.

Early temporal models for pitch (Meddis and Hewitt, 1991a,b; Cariani and Delgutte, 1996a,b), estimated pitch by finding the first major peak in the ACF, or summary autocorrelation function (SACF), but more robust estimations are obtained from more recent models (Cariani, 2004; Bidelman and Heinz, 2011) that incorporate multiple peaks. Using patterns of multiple peaks can reduce octave errors and make successful pitch predictions for a wider range of stimuli. The edge pitch matching data also require multiple autocorrelation peaks (Fig. 1). Edge pitch experiments can reveal the number of peaks that contribute to pitch perception as a function of frequency range.

The more recent models also incorporate more realistic physiology. In order to handle pitch multiplicity—hearing out multiple pitches from double vowels, musical dyads and triads—the autocorrelation process needs to be preceded by cochlear filtering and rectifying neural transduction. Bandpass filtering and half-wave rectification of a temporally detailed waveform avoids cancellations between peaks and valleys within the SACF. The noise band with a sharp spectral edge, as treated in the present paper, is simpler, and useful predictions can be made with an autocorrelation model based only on the average stimulus power spectrum. The only oscillations in the ACF are from the edge itself, and they provide similar information in every off-frequency tonotopic region. For spectral-edge stimuli, the ACF is simple enough that an elementary model, accepting all the peaks without regard for their heights, can be used.

Inspired by Békésy's 1963 report of pitches at both edges of a noise band, Small and Daniloff (1967) studied the pitches of LP and HP noise. They initially considered a sine-tone pitch matching experiment, similar to the experiment reported here, but gave it up as “time consuming and difficult for subjects.” Instead, they asked subjects to adjust a filter to produce an edge pitch that was an octave above or below a standard noise having an edge. Despite the difference in methods and the very shallow slopes used by Small and Daniloff (1967; ±35 dB/octave), there are a number of parallels between their results and ours. Their subjects were unable to hear reliable edge pitches for HP noise with edge frequencies (*f_e_*) below 610 Hz, but subjects had no trouble with LP noise having much lower edge frequencies. That result resembles our experience with low *f_e_*, though our HP limit was about two octaves lower than 610 Hz, probably because of our sharper edge.

Small and Daniloff (1967) had ten listeners, and five of them were able to attempt octave matches above a 9620-Hz HP edge. Three of them attempted octave matches above a 9660-Hz LP edge. Evidently they too had some listeners, like our listener *S*, who were capable of hearing edge pitch well above the 5-kHz limit expected for neural timing.^7^ Apparently they, too, found HP noise easier than LP noise for high edge frequencies.

For both LP and HP noise, Small and Daniloff (1967) found that attempts to match an octave above a standard were too high (matching edge frequency more than a factor of 2 greater than standard edge frequency) and attempts to match an octave below a standard were too low. Given the increasing magnitudes of the relative pitch shifts observed in our experiments as the frequency decreases and the signs of those pitch shifts, we would have predicted their results for HP noise but the opposite to their results for LP noise. Possibly the comparison is frustrated by the well-known octave enlargement (Ward, 1954).

Pitches evoked by sharp spectral edges have been studied through pitch matching experiments on periodic complex tones with many strong harmonics (Martens, 1981; Kohlrausch and Houtsma, 1992). Such complex tones include multiple pitch cues—the low pitches of the complex and the pitches of resolved or partially resolved components. The stimulus is more complicated than noise bands. Kohlrausch and Houtsma (1992) found that pitch matching variance monotonically decreased as the fundamental frequency of the complex decreased, increasing the spectral density. By extrapolation, one might expect the smallest variance for noise bands—the ultimate in spectral density. These authors also found that the pitch of a lowpassed complex tone with a 2000-Hz edge was usually matched by a sine tone *above* 2000 Hz. This is also the prediction of a periodicity analysis of the waveform (Hartmann, 1998). The stark contradiction between that observation and the unambiguous evidence that edge pitches occur below the edge frequency for LP noise, as well as the complexity of the complex tone stimulus, discourages attempts to unify these two edge pitch effects.

## VIII. SUMMARY

Open-set melodic interval identification experiments show that NEPs qualify as musical pitches for both LP and HP noise, although there are pitch shifts away from the edge frequency (*f_e_*). The pitch shifts found by Klein and Hartmann (1981) were similarly found in the experiments reported here using higher quality digital stimulus generation. Specifically, the pitch of a LP noise is below *f_e_* and the pitch of a HP noise is above *f_e_*. These pitch shifts were helpful data in evaluating models of edge pitch perception: a temporal model, and a place model.

### A. Temporal model

The temporal model of NEP hypothesized that pitch is determined by a characteristic autocorrelation lag, which is a mean of the weighted lags of the peaks of the broadband noise ACF. Parsimony was served by approximating the ACF by a sinc function, corresponding to a rectangular power spectrum. This model makes several predictions in agreement with experiments: (1) The predicted sign of the pitch shift agrees both for LP and for HP noise. (2) The pitch shift magnitude is larger, as a percent of the *f_e_*, for lower *f_e_*. (3) The pitch shift magnitude is larger for HP than for LP. (4) The pitch prediction as a function of *f_e_* has about the right curvature. (5) The window duration, which is the adjustable parameter in the temporal model, is within a reasonable range. (6) The optimum window duration increases with decreasing *f_e_* as expected.^8^

### B. Place model

An alternative model for NEP is based on a place theory of pitch perception in which the edge of the excitation pattern is sharpened by lateral inhibition. In this model, tonotopically ordered neurons at some level of the auditory system are inhibited by excitation of neighboring neurons with higher and lower characteristic frequencies. At an edge, the primary excitation of neighbors beyond the edge disappears and so does their ability to inhibit excitation. The result is an enhancement of excitation of neurons near the edge that *do* receive primary excitation. The enhancement therefore occurs at places having characteristic frequencies below the edge frequency for lowpassed noise and above the edge frequency for HP noise, in agreement with experimental pitch shifts. Predicting the pitch shift requires an estimate of the displacement of the peak of the enhancement away from the edge. If the displacement is a constant fraction of the edge frequency (Shamma, 1985) the predicted pitch shift would be a flat line on a pitch shift plot such as Fig. 3(b). If the displacement is related to neural coordinates as initially established in the cochlea (e.g., Greenwood, 1961), the relative displacement increases for decreasing frequency—a behavior that is consistent with the experimental observations of edge pitch shifts as shown in Fig. 3(b).

### C. Critical experiments

Experiments using low and high edge frequencies were done to try to distinguish between the temporal and place models.

#### 1. Low edge frequency

The experiments of Sec. IV showed that as the edge frequency decreased below 150 Hz, the pitch persisted for a LP noise but disappeared for HP noise. As argued in Sec. IV C, this result is consistent with the timing model but not with a place model that assumes asymmetrical auditory filters.

#### 2. High edge frequency

The experiments of Sec. VI showed that for high edge frequencies, the pitch was stronger for HP noise than for LP noise. As argued in Sec. VI C, this result is consistent with both timing and place models.

As the edge frequency approached 5000 Hz, the pitch disappeared for most listeners, but for at least one listener a pitch persisted up to an edge frequency of 10 000 Hz. The latter result argues against a timing model. The temporal model for NEP requires that neural synchrony be maintained in the frequency region of the edge. If useable neural synchrony disappears as the stimulus frequency passes 5 kHz, then the edge pitch ought to disappear as well. Shamma's 1985 lateral inhibition model also requires synchrony. Experiments with both LP and HP noise found that edge-pitch matching deteriorated considerably above 5 kHz for listeners *A*, *B*, *I*, and *Z*. However, listener *S* made matches with a standard deviation of less than a semitone for an 8-kHz LP edge and also for an 11-kHz HP edge. Further, although the matches by the other listeners may have been inaccurate for edge frequencies near 5 kHz, their data do not support the conclusion that the edge pitch disappeared entirely. Either temporal synchrony is, at least weakly, maintained at high frequencies in human listeners or listeners are able to exploit place of encoding to hear an edge pitch. Place of excitation as enhanced by lateral inhibition at an edge might provide some high-frequency information for all listeners and lots of information for listener *S*.

## Figures and Tables

**FIG. 10.**
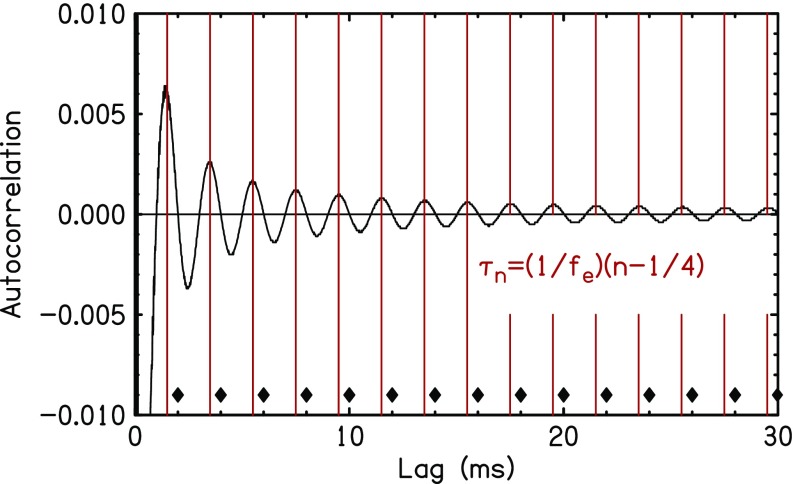
(Color online) Autocorrelation for a HP noise band with equal-amplitude, random-phase components from 500 to 20 000 Hz. It is normalized to a value of 1.0 (well off the plot) for a lag of zero. The vertical lines are drawn at lag values given by *τ_n_* = (*n* – 1/4)/*f_e_*, *f_e_* = 500 Hz, for 1 ≤ *n* ≤ 15 as a test of the *n* – 1/4 approximation for the lag values of the peaks. Close inspection reveals a discrepancy for the leftmost peak, but discrepancies for the other peaks are too small to be seen. Diamonds indicate integer multiples of the period 1/*f_e_*. They are one-quarter period away from the vertical lines.

**FIG. 11.**
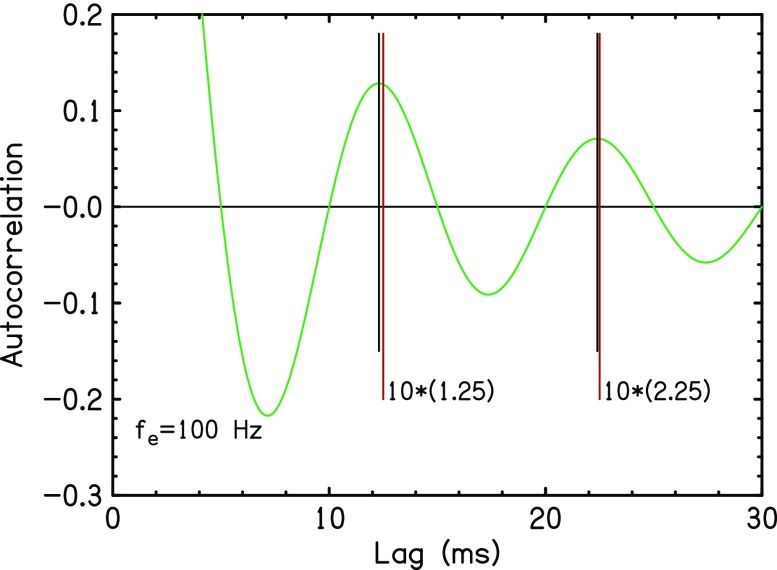
(Color online) Sinc function for a LP edge at *f_e_* = 100 Hz. Two vertical lines are drawn for each peak, the shorter at the exact peak for the sinc function, the longer at the approximate lag value (*n* + 1/4)/*f_e_*. The long and short vertical lines become indistinguishable as either the lag increases or the frequency increases (or both).

## Footnotes

^1^ The 1981 experiment used only three different digital sound files with edge frequencies in three ranges. Finer gradation of edge frequencies was obtained by changing the sample rate. Because the edge frequencies from different ranges overlapped, small shifts attributable to details of the sound files themselves were observed, but the data from different ranges are combined in Figs. 2 and 3 for simplicity. The different ranges can be seen in the 1981 publication.

^2^ Lateral inhibition, if it exists anywhere in the auditory system, might occur at any level of the auditory neural pathway. An inhibition model based on the Greenwood formula for the cochlea makes the assumption that tonotopy at the site of the putative lateral inhibition follows the tonotopy in the cochlea. Therefore, place shifts at the relevant site can be represented by displacements along the cochlear partition as measured in millimeters.

^3^ The experiments by Klein and Hartmann (1981) maintained a broadband noise throughout an experimental trial, including the nominally “silent” intervals. It was thought that including a noise background (without an edge) along with the matching sine tone would benefit the listener by making the matching interval sound more like the target interval. Subsequent experience with edge pitch matching indicated no perceived benefit from the noise background and none was used in the current experiments.

^4^ The consistency in matches for listener *S* can be understood as a proclivity to match indistinguishable edges by sine tones near 50 or 60 Hz. For an edge at 100 Hz, 13 of 16 matches were closely spaced and too low (average –884 cents) pointing to 60.0 Hz. At 70 Hz, 11 of 16 matches were too low by an average of –333 cents and four others were an octave above that. These matches pointed to 57.8 Hz. At 50 Hz, 10 of 16 matches were too high by an average of 198 cents, and the other 6 matches were an octave above that. These matches pointed to 56.0 Hz. Therefore, the preponderance of matches (means, 60.0, 57.8, and 56.0 Hz) were made at about the same frequency for the low edge-frequency range, independent of what the edge frequency actually was.

^5^ The autocorrelation model for a HP noise predicts that the pitch for a 1200-Hz edge should be higher than for a 600-Hz edge by a factor of 1.96 (30-ms window) or 1.94 (15-ms window) instead of 2.00. Both ratios are considerably higher than the equal-tempered minor 7th at 1.78. If the perceptual octave above 600 Hz is enlarged by 1% per McKinney and Delgutte (1999), i.e., 1212 Hz, a minor 7th could correspond to a slightly larger ratio (1.8), but this ratio is still much smaller than 1.94.

^6^ Data from the last 12 runs of this initial series showed 11 of 12 matches at *f_e_* = 6 kHz within ±50 cents of the edge (12 of 12 with octave correction). At 7 kHz, 11 of 12 matches were within 100 cents of the edge frequency (with one octave correction). At 8 kHz, 11 of 12 matches were within 150 cents of 8 kHz.

^7^ In connection with unexpected results, it may be worth noting the caveat about octave switching in the filter as suggested by Small and Daniloff (1967).

^8^ The experiment protocol included trial-by-trial feedback to the listeners intended to maintain listener interest. The listeners knew that some pitch shift was an expected result, which would discourage them from adjusting their responses to reduce the shift. Also, the listeners were so skilled at the matching task, and the experiments continued for so many trials, that we think it unlikely that the feedback had a significant effect on the data. Nevertheless, some bias may have occurred, especially because most of the experiment runs were blocked—LP noise or HP noise. If it had occurred, bias would likely have reduced the measured pitch shift. Such reduction would have had the effect of artificially increasing the estimates of the autocorrelation window duration.

## APPENDIX A: PREDICTIONS OF THE SINC-AUTOCORRELATION MODEL

The autocorrelation model of pitch predicts pitch based on the pattern of peaks as a function of the autocorrelation lag, the temporal variable. For example, for a periodic complex tone, the major peaks occur at lag values that are exactly the periods of the tone (*T*,2 *T*,3 *T*,…). For a noise band with a sharp edge, the peak pattern is different, and the observed pitches provide evidence about just what aspect of the peak pattern is relevant for pitch.

This Appendix shows how the peaks of a model ACF can be used to predict the pitches of LP and HP noise bands with sharp edges. It leads to formulas, Eqs. (3) and (4), that are used to make all the pitch predictions for the temporal model.

### 1. LP

If the peaks of an ACF occurring at lag values *τ_n_* are approximately equally spaced along the lag axis, as for the sinc-ACF of Eq. (1), each *τ_n_* value can be associated with an integer multiple of a dominant periodicity $\hat{\tau }$. The dominant periodicity points to a pitch *p*, $p=1/\hat{\tau }$. Thinking this way, we might choose the best $\hat{\tau }$ to minimize the error *E*_1_,

$$E_{1}=\sum_{n=1}^{N}{\left(\tau_{n}-n\hat{\tau }\right)}^{2}.$$ (A1)

Setting the derivative of *E*_1_ equal to zero leads to

$$\hat{\tau }=\frac{6}{N(N+1)(2N+1)}\sum_{n=1}^{N}n\tau_{n}.$$ (A2)

This equation makes intuitive sense if we imagine that *τ_n_* ≈ *n*/*p*, in which case we obtain the consistent result

$$\hat{\tau }\approx \frac{6}{N(N+1)(2N+1)}\frac{1}{p}\sum_{n=1}^{N}n^{2}=\frac{1}{p}.$$ (A3)

However, the calculation of $\hat{\tau }$ from Eq. (A2) is counter intuitive in that this equation gives increasing weight to peaks of longer lag (i.e., larger *n*). Therefore, we abandon the minimization of *E*_1_ in Eq. (A1). Instead we imagine that the lag of each peak beyond the first represents the period of a subharmonic. In the context where the peaks are approximately equally spaced, the *n*th peak points to a frequency that is the reciprocal of *τ_n_*/*n*. Thinking this way, we choose the best $\hat{\tau }$ to minimize the error *E*_2_,

$$E_{2}=\sum_{n=1}^{N}{\left(\tau_{n}/n-\hat{\tau }\right)}^{2}.$$ (A4)

The best estimate of $\hat{\tau }$ then becomes

$$\hat{\tau }=\frac{1}{N}\sum_{n=1}^{N}\tau_{n}/n.$$ (A5)

For the LP sinc-ACF, the lag values of the peaks are given by the approximation in Eq. (2), τ_*n*_ = (*n* + 1/4)/*f_e_*. This approximation is tested in Appendix B. Therefore,

$$\hat{\tau }=\frac{1}{N}\sum_{n=1}^{N}\left[1+1/(4n)\right]/f_{e}=(1/f_{e})\left\{1+\left[1/(4N)\right]\sum_{n=1}^{N}\left(1/n\right)\right\}.$$ (A6)

The sum ∑1/n is called a “harmonic series” by mathematicians (because of the wavelengths of successive harmonics in a periodic complex tone), and it is approximately equal to ln(*N*) + *γ* + 1/(2 *N*), where *γ* is the Euler-Mascheroni constant, *γ* ≈ 0.57722.

The reciprocal of $\hat{\tau }$ is the predicted value of pitch *p*. Therefore the ratio of the pitch to the edge frequency for LP noise is

$$p/f_{e}=\frac{1}{1+[1/(4N)][\ln(N)+\gamma +1/(2N)]}.$$ (A7)

Equation (A7) is Eq. (3) in the text.

### 2. HP

The ACF for a rectangular HP noise band, *a*_HP_, can be computed by starting with the ACF for an infinite band, having power at all frequencies from zero out to infinity (*a*_∞_), and then subtracting the ACF for a LP rectangular band,

$$a_{\text{HP}}(\tau )=a_{\infty }(\tau )-a_{\text{LP}}(\tau )=a_{\infty }-\text{sin}(2\pi f_{e}\tau )/(2\pi f_{e}\tau ).$$ (A8)

Function *a*_∞_ is a delta function, and its area can be chosen to fulfill the usual normalization requirement for ACFs, *a*_HP_(0) = 1. Because of the minus sign in Eq. (A8), *a*_HP_(*τ*) has peaks where *a*_LP_(*τ*) has valleys. The two functions differ by half a period. For the sinc-ACF, the peaks of *a*_HP_(*τ*) occur at *τ_n_* = (*n* – 1/4)/*f_e_*, which is just the same as Eq. (2) except for the minus sign. It follows that the pitch prediction for HP NEP is the same as Eq. (3) [or Eq. (A7)] for LP NEP except for a sign, namely

$$p/f_{e}=\frac{1}{1-[1/(4N)][\ln(N)+\gamma +1/(2N)]}.$$ (A9)

This equation is Eq. (4) in the text.

Because it is calculated from the components that are missing from the spectrum instead of the components that are present in the spectrum, the ACF for HP noise, *a*_HP_, is less well defined than the ACF for LP noise. The rest of this Appendix is a test to examine *a*_HP_, especially the peak locations, with a numerical example.

Figure 10 is the normalized autocorrelation for a noise with equal-amplitude, random-phase components from 500 to 20 000 Hz. Like the experimental stimuli, the spectrum was attenuated between 16 and 20 kHz, the duration was 500 ms, and the sample rate was 100 000 sps. Figure 10 shows that the peaks of the envelope agree reasonably with the *n* – 1/4 approximation noted just below Eq. (A8), and that was an essential reason for the test. However, the actual values of autocorrelation are small because the function is normalized by all the components in the band. Restricting the band to the region where neural synchrony occurs or to a single auditory filter would increase the size of the normalized function.

## APPENDIX B: THE ONE-QUARTER SHIFT APPROXIMATION

The autocorrelation peak model derived in Appendix A hypothesizes that pitch is determined by a weighted average of the lag times corresponding to peaks of the ACF. Equations (3) (LP) and (4) (HP) compute predicted pitches based on approximate peak locations using the “*n* ± 1/4” approximation. The *n* + 1/4 approximation for LP noise was tested by recomputing predicted pitches based on exact peak locations of the sinc function. An example sinc function with the discrepancy indicated is shown for a LP edge at 100 Hz in Fig. 11. For edge frequencies higher than 100 Hz, the discrepancy is even smaller and harder to see.

The test was run for frequencies from 50 to 1000 Hz by calculating predicted pitch shifts, defined as the ratio of predicted pitch to edge frequency. The error in pitch shift caused by the *n* + 1/4 approximation was largest for 50 Hz and decreased monotonically for increasing frequency. For 50 Hz the exact ratio was 0.813 and the approximate ratio was 0.788, an error of −3.1%. But as the frequency increased to 150 Hz, the exact ratio was 0.8908, the approximate ratio was 0.8845, and the error was −0.7%. For HP noise, used in the experiments, the error was −1.3% at 150 Hz and −0.9% at 200 Hz.
